# Gene Co-Expression Networks Restructured Gene Fusion in Rhabdomyosarcoma Cancers

**DOI:** 10.3390/genes10090665

**Published:** 2019-08-30

**Authors:** Bryan R. Helm, Xiaohui Zhan, Pankita H. Pandya, Mary E. Murray, Karen E. Pollok, Jamie L. Renbarger, Michael J. Ferguson, Zhi Han, Dong Ni, Jie Zhang, Kun Huang

**Affiliations:** 1 Department of Medicine, Indiana University School of Medicine, Indianapolis, IN 46202-3082, USA; 2 National-Regional Key Technology Engineering Laboratory for Medical Ultrasound, Guangdong Key Laboratory for Biomedical Measurements and Ultrasound Imaging, School of Biomedical Engineering, Health Science Center, Shenzhen University, Shenzhen 518060, China; 3 Department of Pediatrics, Indiana University School of Medicine, Indianapolis, IN 46202-3082, USA; 4 Department of Pharmacology and Toxicology, Indiana University, Indianapolis, IN 46202-3082, USA; 5 Department of Medical and Molecular Genetics, Indiana University School of Medicine, Indianapolis, IN 46202-3082, USA; 6 Regenstrief Institute, Indianapolis, IN 46202, USA

**Keywords:** rhabdomyosarcoma, gene fusion, gene co-expression analysis, quasi-clique merger, copy number variation

## Abstract

Rhabdomyosarcoma is subclassified by the presence or absence of a recurrent chromosome translocation that fuses the *FOXO1* and *PAX3* or *PAX7* genes. The fusion protein (FOXO1-PAX3/7) retains both binding domains and becomes a novel and potent transcriptional regulator in rhabdomyosarcoma subtypes. Many studies have characterized and integrated genomic, transcriptomic, and epigenomic differences among rhabdomyosarcoma subtypes that contain the *FOXO1-PAX3/7* gene fusion and those that do not; however, few investigations have investigated how gene co-expression networks are altered by *FOXO1-PAX3/7*. Although transcriptional data offer insight into one level of functional regulation, gene co-expression networks have the potential to identify biological interactions and pathways that underpin oncogenesis and tumorigenicity. Thus, we examined gene co-expression networks for rhabdomyosarcoma that were *FOXO1-PAX3* positive, *FOXO1-PAX7* positive, or fusion negative. Gene co-expression networks were mined using local maximum Quasi-Clique Merger (lmQCM) and analyzed for co-expression differences among rhabdomyosarcoma subtypes. This analysis observed 41 co-expression modules that were shared between fusion negative and positive samples, of which 17/41 showed significant up- or down-regulation in respect to fusion status. Fusion positive and negative rhabdomyosarcoma showed differing modularity of co-expression networks with fusion negative (*n* = 109) having significantly more individual modules than fusion positive (*n* = 53). Subsequent analysis of gene co-expression networks for *PAX3* and *PAX7* type fusions observed 17/53 were differentially expressed between the two subtypes. Gene list enrichment analysis found that gene ontology terms were poorly matched with biological processes and molecular function for most co-expression modules identified in this study; however, co-expressed modules were frequently localized to cytobands on chromosomes 8 and 11. Overall, we observed substantial restructuring of co-expression networks relative to fusion status and fusion type in rhabdomyosarcoma and identified previously overlooked genes and pathways that may be targeted in this pernicious disease.

## 1. Introduction

### 1.1. Gene Fusions in Solid Tumors

Gene fusion is an important consequence of mutation that has been observed in many cancers including malignant solid tumors, leukemia, and especially pediatric sarcoma [1,2]; such fusions may be drivers of, or contribute to cancer progression. Advances in next generation sequencing technologies have empowered a new generation of genomic, transcriptomic, and epigenetic studies of different cancers, and such studies have highlighted the prevalence of structural variation that lead to gene fusions. Recent studies have underscored the important functional roles of gene fusions in sarcoma biology with the promise of providing insights that lead to treatment and improved prognosis. As an example, rhabdomyosarcoma frequently contain recurrent gene fusions involving *FOXO1* genes and *PAX* genes amid a changing landscape of genome mutations and structural variations [3]. The presence of gene fusions reduces patient prognosis because they are associated with aggressiveness of the tumors. While some differential expression analyses have been conducted for gene fusions in pediatric sarcomas [4], relatively few systems biology analyses have been conducted for gene fusions in pediatric sarcomas. The goal of this paper is to implement a systems biology approach for the *FOXO1-PAX3/7* fusion in rhabdomyosarcoma using gene correlation network analysis to infer novel biological insights and hypotheses about this aggressive pediatric cancer.

### 1.2. Rhabdomyosarcoma and the *FOXO1-PAX3/7* Fusion

Rhabdomyosarcoma is an aggressive malignancy of soft-tissue that may occur in any non-boney tissue but predominantly arises from striated muscle or myogenic progenitors [5,6,7,8,9,10]. It is a pediatric cancer with most cases occurring in young patients rather than adults [5,6,7,8,9,10]. Rhabdomyosarcoma may occur in a diverse range of tissues, several subtypes of which are recognized based on histopathology and presentation of the disease, including botryoid embryonic, spindle embryonic, alveolar, and anaplastic [5,11]. In the aggressive alveolar-type rhabdomyosarcoma, a translocation occurs in the majority of patient cases that results in a fusion of fork-head box 1 (*FOXO1*) and paired box 3 or paired box 7 (*PAX3/7*) genes [12,13]. The presence of this fusion has been associated with differences in epigenetic regulatory elements [4,14,15,16,17], chromatin content and instability [3,18], copy number variance and structural variation [3,19,20], and gene expression [4,21,22,23]. FOXO1-PAX3/7 fusion positive tumors have higher mortality fusion negative, and diagnosis can lead to improved risk stratification ([24,25,26], but see [27]). *FOXO1* and the *PAX* complex of genes encode transcriptional regulation proteins for cell cycle, growth, and development, and their biology may be an important component of FOXO1-PAX3/7’s effects. It is important to note that the histopathological subclassification of rhabdomyosarcomas may be confounded by molecular differences and vice versa. Classically, alveolar rhabdomyosarcoma are differentiated into *FOXO1-PAX* positive vs. negative; however, embryonic rhabdomyosarcoma are sometimes included with fusion negative alveolar rhabdomyosarcoma, and there is some evidence that these subtypes are indistinguishable clinically [16]. Here, we adopt fusion negative and fusion positive terminology that denotes the presence or absence of FOXO1-PAX3/7 and is agnostic to histopathological designation as the presence or absence of the fusion is integral to understanding how it may change gene correlation networks.

The fusion protein (FOXO1-PAX3/7) contains the activation domain of *FOXO1* and full binding domain of *PAX3* or *PAX7* ([12], reviewed in [28]). DNA binding domains are retained in the fusion protein, which promotes DNA binding and altered gene expression. The fusion protein is expressed and shows strong effects on transcriptional regulation such as (1) PAX3/7 promoted transcription sites become expressed when otherwise silenced early in development; (2) expression is amplified; and (3) the fusion protein localizes to the nucleus ([12], reviewed in [28]). In combination, FOXO1-PAX3/7 becomes a potent transcription regulator that induces downstream changes in gene expression for multiple pathways, including *PAX3/7* targets involved in myogenic differentiation, interference with normal *FOXO1*-signaling, cellular proliferation, apoptosis, and the interplay among them [7,12,17,28,29,30,31,32,33,34]. Presence of the fusion protein alone is insufficient to cause oncogenesis, but it contributes to tumorigenicity and pathogenesis ([28,29] but see [35]).

### 1.3. Gene Co-Expression Networks and Rhabdomyosarcoma

Many studies have documented changes in gene expression with respect to FOXO1-PAX3/7, including high-throughput expression arrays, manipulation FOXO1-PAX3/7 expression in animal models and cell lines, and integrated -omics based approaches [4,12,15,16,21,23,28]. These studies identified key pathways and processes in rhabdomyosarcoma that may be targeted in treatment, *RAS/MEK/ERK* pathway in particular [3,6,15,36]. Recently, Sun et al. [4] conducted a comparison of methylation patterns and gene expression for rhabdomyosarcoma with and without FOXO1-PAX3/7. They observed 548 up- and 454 down-regulated genes in FOXO1-PAX3/7 fusion positive samples based on differential expression analysis and a minimum absolute fold change larger than 2. While differential analysis can reveal how gene expression is affected by fusion status, it is often difficult to infer key biological processes or events without also accounting for the relationships among genes. More importantly, co-expression network analysis can identify statistical associations among genes that have not been previously identified by targeted studies and reduce complexity for better biological inference. Inferring both core and differential biological processes by examining their correlations among expressed genes will almost certainly lead to new hypotheses for these rare, understudied cancers and their molecular subtypes.

### 1.4. Study Overview

For the above reasons, we examined gene co-expression networks in rhabdomyosarcoma with respect to the presence or absence of the FOXO1-PAX3/7 fusion. Gene co-expression network analysis has been widely adopted in bioinformatics to infer gene relationships, predict new gene functions, discover important biological processes, and identify biomarkers for diseases [37,38,39,40,41,42]. Each gene is treated as a node and among gene correlation coefficients—or a transformation of them—can be calculated and used to weight edges linking genes in the network. Such networks may be mined to define clusters of genes with especially strong co-expression, defined as gene modules and summarized as eigengenes, and enable discovery of genes with highly correlated expression in different conditions. Strongly correlated gene modules within correlation networks often share common biological functions or regulation, e.g., participating in the same biological process or pathway and/or co-regulated by the same transcription factors. The biological implications of gene co-expression modules can often be inferred from gene ontology enrichment analysis. In addition, gene module co-expression can be used for comparative analysis among network modules or in response to different conditions with much greater statistical inference; e.g., in response to fusion status and type. With these potential advantages, we examined the gene correlation networks of gene expression microarrays from the Sun et al. [4] study of fusion negative, FOXO1-PAX3 positive, and FOXO1-PAX7 samples. We identified and compared gene correlation networks as a response to the presence/absence and type of fusion for rhabdomyosarcomas. We hope this translational bioinformatics study leads to new insights about the underlying systems biology, as well as a confirmation of existing knowledge about this pernicious pediatric disease.

In addition to being functionally related, co-expressed genes could also reside on the same cytoband of a chromosome. This is particularly common in cancer samples. Such enrichment of co-expressed genes on specific cytobands often implies copy number variation (CNV) events on these cytobands among the patients. Due to structure proximity, expression levels of genes affected by the same CNV event can be correlated. Given the prevalence of CNV events in cancers, we can also take advantage of gene co-expression network mining to identify potential CNV events. We compared gene co-expression modules that were differentially expressed relative to fusion with CNV information from four new rhabdomyosarcoma cases at Riley Children’s Hospital (Indianapolis, IN, USA). We observed that co-expression modules overlapped significantly with genes that also had copy number variants, especially those observed in patients that were FOXO1-PAX3/7 negative. These fusion positive patients harbored fewer copy number variants, whereas fusion negative harbored many, which was consistent with previous studies of copy number variance in rhabdomyosarcoma [3,6,8,15,18].

## 2. Materials and Methods

### 2.1. Data Source and Pre-Analysis Processing

Gene expression data of rhabdomyosarcoma tissue from 25 patients with fusion-negative type, 26 patients with FOXO1-PAX3 type, and 7 patients with FOXO1-PAX7 type were obtained from NCBI Gene Expression Omnibus (GSE #66533). The determination of fusion status and type are described by Sun et al. [4] and details therein [43]. GEO data was downloaded as robust multiarray average (RMA) normalized probe expression values from an Affymetrix GeneChip Human Genome U133 Plus 2.0 microarray (Thermo Fisher Scientific Inc., Waltham, MA, USA). Gene expression series data were imported into R using the GEOquery package [44] from BioConductor [45].

The raw data contained 54,675 probes mapped to ~35,000 gene symbols; however, multiple probes were matched to the same gene. Probes without gene symbols were removed, and the maximum probe value was used as the gene expression value. Co-expression analysis is based on gene-by-gene regressions which require variance and covariance for meaningful inference [42,46]. For this reason, the genes with expression variance in the bottom 33% quantile were removed to reduce noise in the co-expression analysis and reduce computational demands while retaining many genes for gene co-expression network (GCN) analysis (*n* = 15,759).

### 2.2. Gene Co-Expression Network Analysis

All data manipulation and statistical methods were performed using R version 3.5.1 [47] and R packages (described below). Gene co-expression network analysis was performed with local maximum Quasi-Clique Merger (lmQCM), using the R package lmQCM [46]; for theory and background see [41,42,46,48]. lmQCM analysis was implemented with *γ* = 0.7, and correlation matrices were calculated using Spearman Rank correlation coefficient.

### 2.3. Relating Co-Expression Module Expression Variance and Fusion Status

The aim of co-expression analysis was to identify co-expressed gene modules that differed with respect to fusion positive and fusion negative samples. To identify which co-expression modules had differential expression with respect to fusion status, we performed principal component analysis and subsequently analyzed projected loading values using permutational analysis of variance with fusion status (positive vs negative and *PAX3* vs. *PAX7*) as an explanatory variable. First, principal components analysis was performed separately for each co-expression module and sample loading values were calculated for each principal component. In this analysis, the first principal component represented the axis of greatest gene expression variation for each module. Next, the among sample distance matrix of loading values were calculated and treated as a response variable using permutational analysis of variance with fusion status as an explanatory variable using the adonis function from the vegan package for R [49]. Co-expression modules were considered as associated with fusion status and type differences when *p* < 0.05. This analysis probes the degree to which gene expression variance is explained by fusion status of co-expressed modules common to all rhabdomyosarcoma samples, i.e., differential expression is implied by a significant effect of fusion status.

A key difference between lmQCM and other gene co-expression network algorithms is that it allows genes to belong to multiple co-expressed modules, i.e., gene overlap is permitted. Genes that are merged into multiple modules may represent genes that integrate related but interactively distinct gene interaction networks. Thus, gene overlap among the 4 largest upregulated and 4 largest downregulated co-expression were identified and described to identify genes that link co-expressed modules.

Gene co-expression analysis was performed on fusion negative and fusion positive samples separately to compare overall metrics of network structure: module count and size. Because fusion positive samples were potentially confounded by two unique *PAX* fusion types with unique GCNs, fusion positive samples were secondarily investigated for differential co-expression between *PAX3* and *PAX7*-type fusions (See Section 2.2 for details). While not a primary aim of the manuscript, we observed that several co-expression modules were differentially regulated between the two fusion types.

### 2.4. Functional Enrichment Analysis

Modules that showed an association between expression variance and fusion status were subsequently analyzed for functional enrichment and co-expression atlas using ToppFunn (https://toppgene.cchmc.org/). Gene ontology (GO), cytoband analysis, and co-expression atlas terms that were considered statistically significant if the false discovery rate (FDR) <0.05. In addition, we performed pathway analysis using Ingenuity^®^ Pathway Analysis (https://www.qiagenbioinformatics.com/) from QIAGEN, Inc. (Venlo, The Netherlands) for gene modules that were significantly associated with *PAX3* and *PAX7* differences from fusion positive network analysis. This was performed in addition to ToppFunn enrichment analysis because these gene modules had low affinity for specific biological processes and co-expression atlas annotations, and such an analysis can highlight additional information about identified gene networks.

### 2.5. Copy Number Variation in Rhabdomyosarcoma

Four rhabdomyosarcoma cancer samples were sequenced using Illumina platform with the data processed using the BWA-SAMBLASTER-GATK pipeline. This analysis generated a VCF file that included copy number alteration and breakpoint coverage for rhabdomyosarcoma samples. Based on the structural variation data, one of the four samples harbored the *FOXO1-PAX3* gene fusion. Based on the copy number alteration data, a circular binary segmentation (CBS) analysis was performed using the R package DNAcopy [50]. Segments were mapped to reference hg19 genome assembly. Gene-level copy number variation (CNV) was estimated using the GISTIC2 method [51]. Genes were categorized as having copy number variation based on when CNV ≥ 0.2 or ≤ 0.2. Genes with CNV values ≤ 0.2 were categorized as deletion and ≥ 0.2 were categorized as amplifications (based on [52]).

The list of genes with significant copy number variation from both fusion negative and positive samples were compared with the genes mapped into co-expression modules (see above). Counts of gene overlap were analyzed using Fisher’s exact test and a *p* < 0.05 for statistical significance.

## 3. Results

### 3.1. Consensus Network Analysis of Fusion-Associated Module Expression Patterns

**Figure 1:**
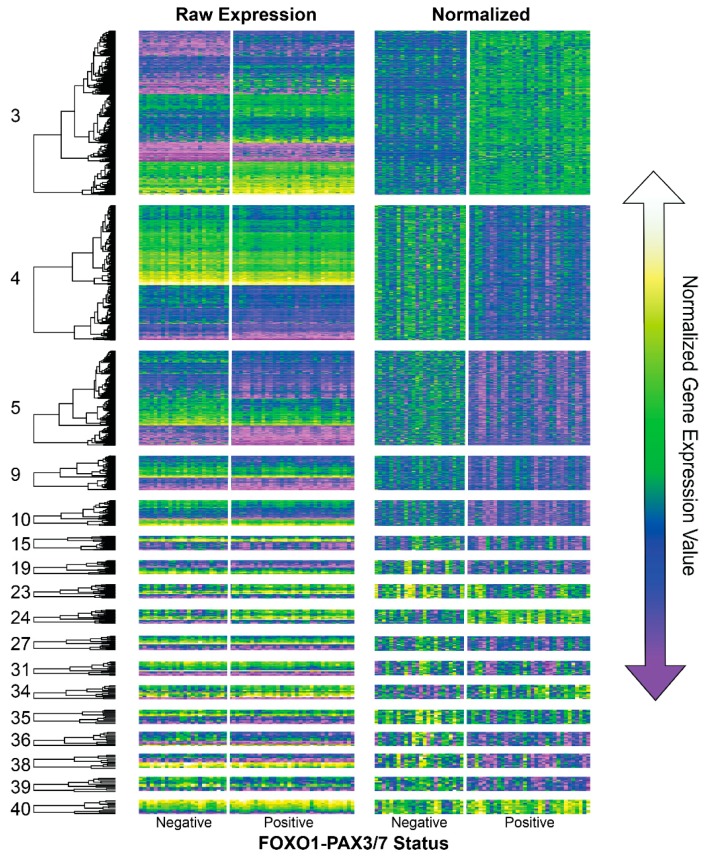
Gene expression heatmaps of co-expression modules that were identified from consensus lmQCM network modeling and that showed differential gene regulation with respect to FOXO1-PAX3/7 fusion status (see Table 1). Expression values have been organized by hierarchical clustering to better visualize absolute expression values (left). To better visualize potential differences between *PAX3* and *PAX7* type fusions, gene expression values were mean centered and normalized from −1 to 1 for each module separately (right).

lmQCM consensus network analysis identified 41 gene co-expression modules that were shared in fusion negative and positive samples (Table S1). A subset of these modules had a statistically significant relationship between fusion status and gene co-expression patterns (Figure 1, Table 1). 4/17 modules contained upregulated genes in FOXO-PAX3/7 positive samples, whereas 13/17 contained downregulated genes (Figure 1 and Figure S1).

**Figure 2:**
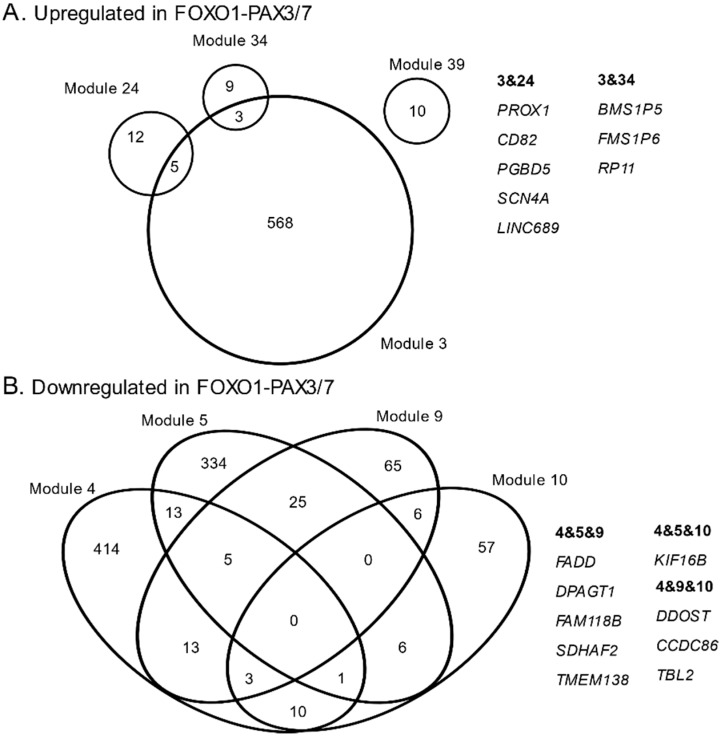
Venn diagrams of gene counts and overlap for co-expression modules that were upregulated (**A**) and downregulated (**B**) in rhabdomyosarcoma samples that were positive for the FOXO1-PAX3/7 fusion.

Modules 3, 24, 34, and 40 showed elevated expression values in FOXO1-PAX3/7 positive samples compared to fusion negative (Figure 1). There was limited overlap among the gene names for these 4 co-expression modules, especially compared with fusion negative networks (Figure 2). 5/17 genes from module 24 and 3/12 genes from module 34 were co-expressed in module 3; however, no other overlaps for these modules were observed (Figure 2A). Overlap genes in modules 3 and 24 were *PROX1*, *CD82*, *PGBD5*, *SCN4A*, and *LINC00689*; overlap genes in modules 3 and 34 were *BMS1P6*, *BMS1P5*, and *RP11-235E17.4*. Of these genes, several may be important for rhabdomyosarcoma, including a homeobox transcription factor (PROX1), a tumor suppressor (CD82), a transposable element (PDBD5), a muscle sodium channel (SCN4A), and a long intergenic non-coding RNA (LINC00689) (see Discussion).

Modules 4, 5, 9, 10, 15, 19, 23, 27, 34, 35, 36, 38, and 39 contained genes that were downregulated in FOXO1-PAX3/7 positive samples compared to fusion negative. Many genes were co-expressed in more than one module (Figure 2B), indicating these modules had substantial overlap compared with fusion positive (Figure 2). Nine genes were observed in at least three of the modules, though no single gene appeared in all four. Genes that overlapped with three of the modules were *FADD*, *DPAGT1*, *FAM118B*, *SDHAF2*, *TMEM138*, *KIF16B*, *DDOST*, *CCDC86*, and *TBL2* (Figure 2). Of genes identified in this manner, *TMEM138* and *FADD* are both important for cell cycle, proliferation, and oncogenesis (see Discussion).

### 3.2. Gene Ontology, Cytoband, and Co-Expression Atlas

Gene names from modules associated with fusion status were submitted to ToppGene functional enrichment analysis. The top gene ontology term and cytoband location for each module are reported in Table 2. Many of the modules did not match to specific gene ontology functions and only 8/17 co-expression modules had statistically significant GO term mapping (Table 2 and Table S2). Gene module 3, which was upregulated in fusion positive samples, returned no biological processes as enriched. Genes from this module were significantly localized to cytobands on chromosomes 9, 10, and 16. In contrast, downregulated modules 4, 5, 9, and 10—all downregulated in fusion positive samples—returned biological process enrichments for RNA processing, developmental pathways, and Golgi-related pathways. Modules 4, 5, and 9 were strongly associated with cytobands on chromosomes 8 and 11, while module 10 showed localization to chromosome 19.

Co-expression Atlas enrichment analysis revealed that co-expression modules contained genes with known differential regulation due to FOXO1-PAX3/7 fusion and annotations related to cancer more generally (Table 3 and Table S3). Module 3 returned annotations are defined as genes upregulated in rhabdomyosarcoma that harbor or commonly contain the FOXO1-PAX3/7 fusion and 1 annotation that contains genes downregulated with FOXO1-PAX3/7 is knocked down in cell lines. Module 5 returned annotations that are commonly downregulated when the FOXO1-PAX3/7 fusion is present. Interestingly, genes annotated as developmental or related to stem cells were observed among most co-expression modules, and module 4 contained annotations suggesting broader connections to cancer.

### 3.3. lmQCM of Fusion Negative and Fusion Positive Gene Co-Expression Networks

**Figure 3:**
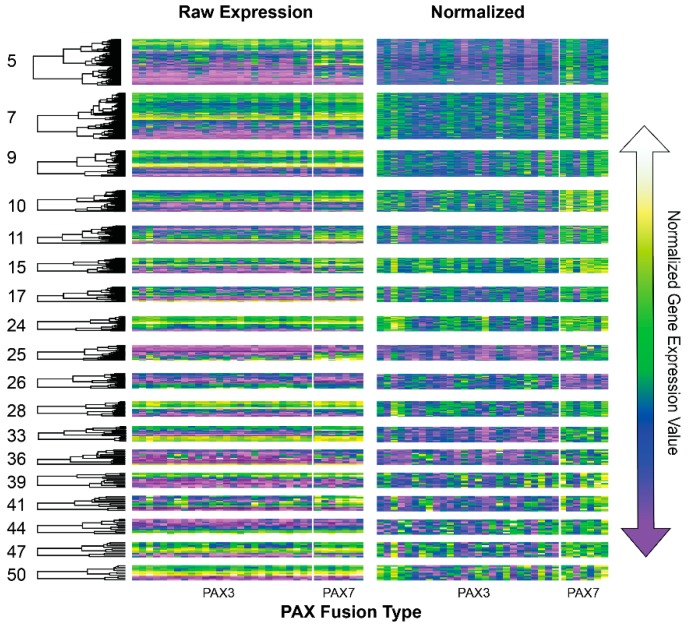
Gene expression heatmaps of co-expression modules that were identified from lmQCM network analysis of FOXO1-PAX3/7 positive samples and that showed differential gene regulation between PAX3 and PAX7 cases. Expression values were organized by hierarchical clustering to better visualize absolute expression values (left). To see potential differences between *PAX3* and *PAX7* type fusions, gene expression values were normalized (right).

We performed lmQCM network analysis on gene expression data on FOXO1-PAX3/7 positive and negative samples separately to assess the degree to which network modularity was affected by fusion. This identified 53 co-expression modules in FOXO1-PAX3/7 positive samples that contained a median of 17 and mean of 138.9 genes. 109 co-expression modules were identified in fusion negative samples with a median of 13 and mean of 70.4 genes. Unexpectedly, several of the co-expression modules from the fusion positive gene correlation network were differentially co-expressed PAX3- versus PAX7-type fusion positive samples (Figure 3). As with consensus network analysis, modules-of-interest were sorted using PCA and permutational Anova analysis. For 17/53 co-expression modules, fusion status—but here, PAX3 vs PAX7 type fusions—caused differential regulation of co-expressed gene modules (Table 4 and Table S4).

**Figure 4:**
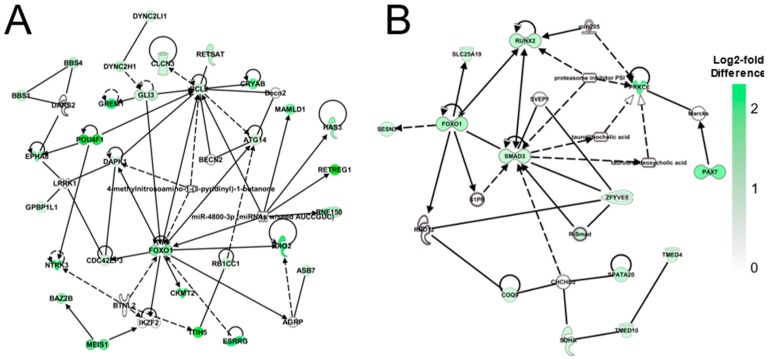
The network visualization of pathway analysis for modules 7 (**A**) and 10 (**B**) from fusion positive network analysis. All genes were upregulated in *PAX7* samples compared to *PAX3*; however, the degree to which genes were upregulated varied. Genes with green coloration had higher log_2_-fold change. Solid lines indicate a direct relationship between two genes, whereas dashed lines indicate an indirect predicted relationship. *FOXO1* and *PAX7* were important genes for both modules and showed 2-fold and 3-fold increase respectively in *PAX7* samples.

We further examined the co-expression modules that were differentially regulated between *PAX3*- and *PAX7*-type and observed that *FOXO1* appeared in modules 7 and 10, which were differentially upregulated in *PAX7* fusions. These genes had expression values that were ~2× higher in *PAX7*- versus *PAX3*-type fusions. In contrast to *FOXO1-PAX3* type fusions, *FOXO1-PAX7* fusions did not associate with co-expression annotations related to prior rhabdomyosarcoma studies when queried using gene enrichment analysis. To better understand how *FOXO1* signaling within these co-expression modules may drive network-level differences in *PAX7*-type, we implemented Ingenuity Pathway Analysis to map known and inferred relationships among these key genes in the presence of the *FOXO1-PAX7* fusion (Figure 4). This analysis revealed that the *PAX7*-specific fusion was associated with an upregulation of BCL2—a critical component of apoptosis (Figure 4A).

### 3.4. Copy Number Variation

We performed whole genome sequencing on four rhabdomyosarcoma samples. One sample was positive for the FOXO1-PAX3/7 gene fusion, and three samples were negative. Copy number variation analysis using a log change value >0.2 resulted in 306 amplifications and 239 deletions for the fusion positive sample. The fusion negative samples had 8746, 7883, 6514 amplifications and 8284, 4214, 1565 deletions, respectively.

### 3.5. Copy Number Variation in Gene Modules from Consensus Network

We examined whether genes with CNV were clustered into one or more co-expression modules from consensus network analysis by comparing overlap. Few co-expression modules with differential expression showed overlapping copy number variation. However, co-expression modules 4 and 5 each had a statistically significant count of genes that also have significant CNV (Table 5). These gene expression modules were significantly associated with multiple cytobands on chromosomes 8 and 11 (Table 2).

## 4. Discussion

We observed co-expressed gene modules that were also differentially regulated with respect to fusion status in rhabdomyosarcoma. Many of the genes that were co-expressed in these modules have been implicated in previous studies of differential expression analyses ([4], especially [22]). Congruence between existing differential expression studies and this network analysis help validate the patterns described and discussed in this study. For example, module 3 contained 139/331 genes upregulated in alveolar compared to embryonic rhabdomyosarcoma, 27/48 genes that were downregulated when FOXO1-PAX3 was altered with RNA interference, and 22/64 genes previously associated with cell line differences related to PAX3 and PAX7 fusions (Table 4 and Table S4). Module 5 contained 75/182 genes that were downregulated in alveolar versus embryonic, and 50/408 genes that were downregulated when FOXO1-PAX3 was overexpressed in mouse models. Because co-expression analysis identifies correlations among genes, genes that have differential expression, but are not strongly correlated with large modules, are not considered here. This could explain why some annotated genes were missing from gene co-expression modules identified in the current study. However, co-expressed genes that were not identified in previous investigations—perhaps due to selection criteria, restrictions of gene expression information, or because relationships among genes were not accounted for—may harbor undiscovered FOXO1-PAX3/7 genes and pathways, which in turn may contain novel targetable pathways for rhabdomyosarcoma.

Many of the gene co-expression modules from consensus analysis also matched with developmental, stem cell, and cancer genes. Modules 4 and 9 contained genes related to leukemia and hepatoblastoma and had significant mapping to chromosomes 8 and 11 (Table 2 and Table 3, Tables S2 and S3). These results suggest that FOXO1-PAX3/7 positive rhabdomyosarcoma show differential expression for genes that are broadly co-expressed in cancers, perhaps representing core growth and proliferative pathways common to oncogenesis. Interestingly, gene enrichment analysis returned developmental and stem cell genes, based on annotations from Davicioni et al. [22]. *PAX* genes are normally a component of embryogenesis, and recent studies have explored the relationship between *PAX* fusions, myogenic differentiation, and muscular phenotypes common in rhabdomyosarcoma [4,6,15,16,17]. These studies have described in detail how activation of myogenic transcription factors downstream of *PAX,* as well as methylation status combine to elucidate the manners in which biological patterns determined by functional mechanisms in rhabdomyosarcoma subtypes. Thus, co-expression modules from our gene correlation network analysis recapitulate known biological phenotypes and mechanisms underlying rhabdomyosarcoma.

A potentially important phenomenon for modeling gene interaction networks is that some genes may interact for two or more co-expression modules. lmQCM is quite different from other gene co-expression network mining algorithms in that it can assign a gene to multiple co-expression modules, which is not the case for hierarchical-based module detection. This helps identify the degree to which different co-expression modules are integrated vs dis-integrated in different conditions. For example, we observed different degrees of overlap in downregulated versus upregulated co-expression modules that were also differentially expressed in fusion negative and fusion positive samples. Upregulated genes tended to act more cohesively as a network with one large module and few small modules that showed low to moderate overlap. This result suggests that co-expression patterns of upregulated genes were driven by stronger correlations that were merged by the lmQCM algorithm.

Importantly, investigating which genes belong to multiple modules may lead to important new biological insights that have been neglected or overlooked by studies that do not permit gene membership in multiple modules. Co-expressed, upregulated genes that were members of multiple modules included a homeobox transcription factor (*PROX1*), a tumor suppressor (*CD82*), a transposable element (*PDBD5*), a muscle sodium channel (*SCN4A*), and a long intergenic non-coding RNA (*LINC00689*). Downregulated modules had co-expression modules with substantially more overlap than upregulated genes (Figure 2). Genes that overlapped with 3 of the 4 largest downregulated modules were identified and included oncogenic genes, e.g., TMEM138, as well as externally mediated apoptosis, e.g., FADD (Figure 2). *FADD* is a particularly important gene to identify in this analysis because it mediates *FAS*-associated cell death and is downregulated along with 3 somewhat distinct co-expression modules. A prior study documented resistance to *FAS*-mediated cell death in alveolar rhabdomyosarcoma cell lines and proposed that targeting the *FAS*-pathway may lead to treatments for rhabdomyosarcoma with gene fusion [53]. Our analysis rediscovered *FADD*.

It is worthwhile to point out that we also tested the widely used Weighted Gene Correlation Network Analysis (WGCNA) R package for GCN module discovery [37,54] and WGCNA generates large modules with hundreds to thousands of genes without being able to detect the smaller GCN modules, suggesting that lmQCM is particularly suitable for discovering small overlapping GCN modules with biological implications. This study is a particularly important and unique demonstration of lmQCM’s ability to assign genes as components of multiple networks and how identification of such genes may lead to important biological insights.

Though we focused on identifying co-expressed gene expression in response to fusion status for this study, lmQCM analysis can also reveal important overall network differences in different conditions. For example, the fusion negative gene correlation network had more, smaller modules compared to fusion positive. This suggests that either gene-level expression correlations were not strong enough to merge smaller modules by lmQCM compared to FOXO1-PAX3/7.

Surprisingly, lmQCM network analysis of fusion positive samples also returned modules that were differentially regulated between *PAX3* and *PAX7* fusions. This is an important contribution to the literature about rhabdomyosarcoma because differences between fusion types are far less studied than fusion presence more generally. Based on gene list enrichment, some of the genes in differentially regulated modules have been reported by prior studies, e.g., 18 genes have been previously associated *PAX3* or *PAX7* expression variance (Table S5). However, most of these genes from differentially co-expression fusion positive modules reported no known responses to *PAX3* or *PAX7* in rhabdomyosarcoma or other cancers. Many, if not most, studies of FOXO1-PAX fusion in rhabdomyosarcoma combine these two fusion subtypes, and few studies have *PAX3* may differ from *PAX7*. Here, we show that *PAX3* vs *PAX7* differences may be widespread to gene co-expression with many of genes having higher expression levels in *PAX7* (Figure 3). For example, *FOXO1* and *PAX7* were both returned as differentially co-expressed in this analysis, which may have important mechanistic explanations for differences between molecular subtypes. Interestingly, *PAX7* fusion caused an upregulation of BCL2—an apoptosis regulator—that was comparatively downregulated in *PAX3*-type fusions. Prior reports have implicated BCL2 as a potential target for pharmaceuticals in fusion positive rhabdomyosarcoma [55]. While further validation of this pattern is necessary, this may suggest that apoptosis pathways have altered expression in fusion subtypes.

Importantly, many co-expression modules were not differentially regulated with respect to fusion status (positive/negative), though there may be graphically distinct patterns that occur among samples (Figure S2). Rhabdomyosarcoma is a diverse syndrome of rare cancers, so many other confounding variables may influence the expression of these co-expressed genes such as stage, tissue of origin, tissue heterogeneity, additional genetic mutations, or other markers of disease progression. Future studies may seek to associate phenotypic, more comprehensive molecular, and clinical data with co-expression modules from these rare diseases. Such investigations may find that disease prognosis, staging, and/or mutational variance are associated with co-expression modules identified in the present study, which were not associated with the FOXO1-PAX3/7 fusion.

## 5. Conclusions

The FOXO1-PAX3/7 fusion results in a novel transcriptional regulator that introduces novel interactions among expressed genes. Prior studies have identified many genes that are differentially regulated with respect the presence or absence of FOXO1-PAX3/7 gene fusion in rhabdomyosarcoma using both experimental and clinical data. But the differential expression analyses that are commonly used to identify fusion biomarkers are agnostic with respect to gene interactions. Here we show that previously implicated genes are frequently co-expressed as networks of genes, raising the issue of whether co-expressed genes are drivers or passengers. Our study importantly rediscovered differential regulation of key apoptotic genes among co-expressed modules that have been previously targeted by pharmaceutical development for rhabdomyosarcoma, though existing literature is limited. Future work may consider how these pharmaceutical agents affect expression for gene co-expression networks as identified in the present study. We also describe many genes that were differentially co-expressed in *PAX3* versus *PAX7* fusions. While the FOXO1-PAX fusion has been well-studied, information about differences between fusion types are generally limited. Here, we provide evidence of hereto unexplored gene interaction networks that differ between fusion types. More importantly, modules described herein provide evidence that FOXO1-PAX3/7 fusion affects not only differential expression of individual genes, but also the interactions among them, which moves beyond the gene-by-gene centric analyses that are commonly conducted. Future work could validate the differences identified by network analysis as a means of discovering and understanding mechanistic differences among fusion subtypes in rhabdomyosarcoma. Implementing a diversity of analytical approaches can lead to exciting, new hypotheses regarding the functional and genetic roles of the FOXO1-PAX3/7 fusion in rhabdomyosarcoma, for which we plan to test experimentally using patient-derived cell and PDX models. Such discoveries will shed light on the development of personalized treatment for this rare, aggressive cancer, and promote similar research in other pediatric cancers.

## Figures and Tables

**Table 1:** Differential co-expression analysis of modules that were significantly different in fusion negative vs positive cases. For brevity, only modules with significant (*p* < 0.05) differences are reported (see Table S1 for full results).

Module	Gene Count	*p*
3	576	0.001
4	459	0.001
5	334	0.001
9	118	0.001
10	84	0.001
15	33	0.005
19	24	0.001
23	17	0.038
24	17	0.001
27	15	0.001
31	13	0.017
34	12	0.001
35	11	0.001
36	11	0.001
38	10	0.001
39	10	0.001
40	10	0.019

**Table 2:** Gene list enrichment analysis of biological processes and cytoband locations 5 largest differentially co-expressed gene modules in fusion negative and fusion positive samples (See Table S2 for all differentially co-expressed modules). Module 3 was upregulated, whereas Modules 4, 5, 9, and 10 were downregulated.

Module	GO ID	Biological Process	FDR B&Y		Cytoband	FDR B&Y
3	NA	NA	NA	|	9q21.11	<0.0001
				|	16p12.3	<0.0001
				|	16p12.2	<0.001
				|	10q26	<0.001
4	6369	RNA Processing	<0.0001	|	8q24.3	<0.0001
	34660	ncRNA metabolic process	<0.0001	|	11q13	<0.0001
	34470	ncRNA processing	<0.0001	|	11q21	<0.0001
	22613	ribonucleoprotein complex biogenesis	<0.0001	| |	8q24.13	<0.0001
	70647	protein conjugation by conjugation or removal	<0.0001	| |	8p21.3	<0.001
5	9790	embryo development	<0.0001	|	11q13	<0.001
	35295	tube development	<0.0001	|	11q21	<0.001
	31175	neuron projection development	<0.0001	| |	11p15.3	0.012
	22008	neurogenesis	<0.0001	|	7q21	0.046
	45595	regulation of cell differentiation	<0.0001	| |		
9	6890	retrograde vesicle transport	<0.0001	|	11q13	<0.0001
	48193	Golgi transport	<0.0001	|	11p15.3	<0.0001
	6888	ER to Golgi vesicle transport	<0.0001	|	11q12.2	<0.001
	30968	endoplasmic reticulum unfolded protein	<0.001	| |	11p15.5-4	<0.01
	6986	response to unfolded protein	<0.001	|	11p12-p11	<0.01
10	6396	RNA Processing	<0.01	|	19p13.3	<0.0001
	22613	ribonucleoprotein complex biogenesis	<0.01	| |	19p13.11	<0.0001
	NA	NA	NA	|	19p13.2	<0.0001
				|	19p13.32	<0.01

Co-expression modules that did not return results from gene enrichment analysis were labeled with the abbreviation “NA” for not available. Processes reported do not directly correspond to cytoband locations reported.

**Table 3:** Top results of ToppGene co-expression analysis for the 5 largest co-expressed modules identified by consensus lmQCM analysis (See Table S3 for more detailed table).

Module	Input #	ID	Name	*p*
3	576	M2012	Genes up-regulated in alveolar rhabdomyosarcoma compared to embryonic rhabdomyosarcoma	*p* < 0.0001
4	475	15902281—Table S1A	Human Leukemia Schoch05	*p* < 0.0001
5	326	M8519	Genes down-regulated in alveolar rhabdomyosarcoma compared to embryonic rhabdomyosarcoma	*p* < 0.0001
9	121	15902281—Table S1B	Human Leukemia Schoch05	*p* < 0.0001
10	80		FacebaseRNAseq e8.5 Hind Brain Neural Epithelium top-relative-expression-ranked 2500 k-means-cluster#2	*p* < 0.0001

**Table 4:** Differential co-expression analysis of modules that were significantly different in *PAX3* vs *PAX7* fusion positive patients. For brevity, only modules with significant (*p* < 0.05) differences are reported (see Table S4 for full results).

Module	Gene Count	*p*
5	178	0.002
7	148	0.006
9	84	0.016
10	72	0.002
11	62	0.001
15	37	0.004
17	32	0.038
24	19	0.03
25	18	0.001
26	18	0.011
28	16	0.015
33	14	0.001
36	13	0.001
39	12	0.004
41	11	0.008
44	11	0.022
47	10	0.045
50	10	0.05

**Table 5:** Count overlap of genes with significant copy number variation and consensus lmQCM co-expression modules that showed statistically significant association with fusion status.

Module	Total Gene Count	Genes with CNV	*p*
3	576	37	0.657
4	459	157	<0.0001
5	334	44	<0.0001
9	118	10	0.241
10	84	4	0.819
15	33	4	0.132
19	24	0	0.398
23	17	0	0.621
24	17	1	1
27	15	0	1
31	13	0	1
34	12	0	1
35	11	0	1
36	11	0	1
38	10	1	0.46
39	10	0	1
40	10	1	0.46
41	10	0	1

Statistical values reported from Fisher’s Exact Test.

## Supplementary Materials

The following are available online at https://www.mdpi.com/2073-4425/10/9/665/s1, Table S1: Differential expression analysis by PCA for co-expressed modules in rhabdomyosarcoma patients with and without FOXO1-PAX3/7 fusion with full report of statistics from permutational Anova, Table S2: Gene list enrichment of biological process and cytoband locations for gene co-expression modules that were differentially regulated in fusion positive versus fusion negative rhabdomyosarcoma, Table S3: Expanded Co-expression Atlas results from gene list enrichment analysis with ToppFunn for 5 largest differentially regulated co-expression modules in fusion negative versus positive rhabdomyosarcoma, Table S4: Differential expression analysis by PCA for co-expressed modules in rhabdomyosarcoma patients with FOXO1-PAX3/7 fusion, Table S5: Top 5 Co-expression Atlas (ToppFunn) results from gene list enrichment for genes that were differentially co-expressed PAX3 versus PAX7 fusion positive rhabdomyosarcoma, Figure S1: The distribution of max gene expression values per probe and within gene expression variance of raw data from GSE 66533, Figure S2: Heatmap plots of normalized gene expression values for 41 co-expression modules that were identified using lmQCM network analysis.
