# Supplementary material for: Gene Co-Expression Networks Restructured Gene Fusion in Rhabdomyosarcoma Cancers
Source: Genes (Basel). 2019 Aug 30;10(9):665. doi: 10.3390/genes10090665 (PMC6770752; doi:10.3390/genes10090665)

Supplemental Figures and Tables

Supplemental Table 1.

Differential expression analysis by PCA for co-expressed modules in rhabdomyosarcoma patients with and without FOXO1-PAX3/7 fusion with full report of statistics from PERMAnova

Module	Gene Count	PC1 Variance Explained	F	R^2	P
1	751	0.400	0.942	0.017	0.346
2	615	0.414	0.180	0.003	0.657
3	576	0.368	278.645	0.833	0.001
4	459	0.451	41.636	0.426	0.001
5	334	0.443	92.984	0.624	0.001
6	201	0.487	0.008	0.000	0.919
7	147	0.578	2.310	0.040	0.143
8	134	0.670	0.008	0.000	0.94
9	118	0.499	36.513	0.395	0.001
10	84	0.505	46.564	0.454	0.001
11	79	0.643	0.014	0.000	0.91
12	72	0.505	3.094	0.052	0.089
13	50	0.619	0.512	0.009	0.515
14	35	0.578	0.755	0.013	0.394
15	33	0.627	6.840	0.109	0.005
16	30	0.593	3.421	0.058	0.078
17	28	0.567	0.509	0.009	0.488
18	24	0.652	1.724	0.030	0.195
19	24	0.639	12.214	0.179	0.001
20	20	0.821	1.232	0.022	0.268
21	19	0.682	3.274	0.055	0.066
22	18	0.617	0.309	0.005	0.602
23	17	0.699	4.702	0.077	0.038
24	17	0.640	39.917	0.416	0.001
25	16	0.601	2.807	0.048	0.102
26	16	0.631	0.093	0.002	0.749
27	15	0.635	14.414	0.205	0.001
28	14	0.628	0.004	0.000	0.942
29	14	0.675	0.058	0.001	0.806
30	13	0.881	1.940	0.033	0.163
31	13	0.696	6.383	0.102	0.017
32	12	0.885	0.152	0.003	0.697
33	12	0.700	1.700	0.029	0.2
34	12	0.685	23.364	0.294	0.001
35	11	0.687	15.395	0.216	0.001
36	11	0.710	18.846	0.252	0.001
37	11	0.682	0.735	0.013	0.415
38	10	0.755	23.875	0.299	0.001
39	10	0.656	25.448	0.312	0.001
40	10	0.659	5.298	0.086	0.019
41	10	0.629	4.297	0.071	0.057

Notes: PERMANOVA was performed on the distance matrix of PC1 with fusion status as the explanatory variable. Significant associations were determined by $P<0.05$.

Supplemental Table 2

Gene list enrichment of biological process and cytoband locations for gene co-expression modules that were differentially regulated in fusion positive versus fusion negative rhabdomyosarcoma

Mod	GO ID	Name	FDR	# Match	Ratio†	Cytoband	FDR	Match Count	Ratio‡
3						10q26	<0.001	5	0.22
4	6396	RNA Processing	<0.0001	73	0.08	8q24.3	<0.0001	28	0.22
5	9790	Embryonic Development	<0.0001	48	0.04	11q13	<0.0001	9	0.08
9	6890	Vesicle Transport, Golgi to ER	<0.0001	10	0.13	11q13	<0.0001	11	0.09
10	6396	RNA Processing	0.0022	16	0.02	19p13.3	<0.0001	11	0.04
15	32963	Collagen Metabolic Process	0.0016	5	0.04	8p21.3	0.019	2	0.07
19						1q24	<0.0001	4	0.07
23	51096	Positive Helicase Activity	0.0035	2	0.33	7p14.1	<0.001	2	0.07
24						3p12.1	<0.0001	2	0.25
27						1p36.1-p35	0.019	1	0.11
31	45040	Protein Import to Mitochondrial Membrane	0.0023	2	0.33	22q13.31	0.0012	2	0.04
34						10q11.22	<0.001	2	0.03
35						1p36.1-p35	0.012	1	0.11
36						19q13.2	<0.001	2	0.08
38						20p12.1	<0.001	2	0.08
39	2000209	Regulation of Anoikis	0.033	2	0.08	20q13.3	<0.001	2	0.1
40						4q31.2-q32.1	0.0029	1	1
41						5q13	<0.001	2	0.09

Notes: Ratio values represent the number of genes matched to a GO term† or cytoband‡ divided by the number of genes

Supplemental Table 3

Expanded Co-expression Atlas results from gene list enrichment analysis with ToppFunn for 5 largest differentially regulated co-expression modules in fusion negative versus positive rhabdomyosarcoma.

Module	Input #	ID	Name	Input #	Annotation #
3	576	M2012	Genes up-regulated in mARMS (molecular ARMS) compared to the mERMS (molecular ERMS) class of rhabdomyosarcoma tumors.	139	331
		16849537-SuppTable2	Human StemCell Davicioni06	132	500
		M7778	Genes down-regulated in Rh4 cells (alveolar rhabdomyosarcoma, ARMS) after knockdown of the PAX3-FOXO1 [GeneiD=5077;2308] fusion protein by RNAi for 72 hr.	27	48
		M4991	'PAX-FKHR signature': genes up-regulated by PAX3- or PAX7-FOXO1 [GeneID=5081;5077;2308] fusion in primary alveolar rhabdomyosarcoma(ARMS) tumors.	25	59
		M12362	Genes up-regulated in RMS cells (rhabdomyosarcoma) expressing PAX3 or PAX7 fusions with FOXO1 [GeneID=5077;5081;2308] compared to the fusion negative cell lines.	22	64
4	475	15902281-TableS1a	Human Leukemia Schoch05	112	449
		M4772	Genes up-regulated in robust Cluster 2 (rC2) of hepatoblastoma samples compared to those in the robust Cluster 1 (rC1).	82	605
		M17936	Genes within amplicon 8q23-q24 identified in a copy number alterations study of 191 breast tumor samples.	47	157
		19061838-TableS7	Human Viral Cairo08	80	801
		M10017	Genes down-regulated in tumor associated macrophages conditioned by: glioblastoma versus colorectal adenocarcinoma	43	197
5	326	M8519	Genes down-regulated in mARMS (molecular ARMS) compared to the mERMS (molecular ERMS) class of rhabdomyosarcoma tumors.	75	182
		16849537-SuppTable2	Human StemCell Davicioni06	73	500
		M19541	Genes commonly down-regulated in human alveolar rhabdomyosarcoma (ARMS) and its mouse model overexpressing PAX3-FOXO1 [GeneID=5077;2308] fusion.	50	408
		M9192	Genes down-regulated in luminal-like breast cancer cell lines compared to the mesenchymal-like ones.	48	450
		20937356-TableS1	Rat Hypothalamic Mansuy10	59	894
9	121	15902281-TableS1b	Human Leukemia Schoch05	47	767
		M2105	Genes whose expression was significantly and positively correlated with the number of perineuronal oligodendrocytes in the layer III of BA9 brain region.	25	756
		M1493	Genes within amplicon 11q12-q14 identified in a copy number alterations study of 191 breast tumor samples.	14	158
		17683608-TableS3	Mouse StemCell Ulloa-Montoya07	22	692
		M5314	Transcripts depleted from pseudopodia of NIH/3T3 cells (fibroblast) in response to haptotactic migratory stimulus by fibronectin, FN1 [GeneID=2335].	21	668
10	80		FacebaseRNAseq e8.5 Hind Brain Neural Epithelium top-relative-expression-ranked 2500 k-means-cluster#2	22	933
			Sample Type by Project: Shred 1/TCGA-Bladder/Urothelial Carcinoma/Muscle invasive urothelial carcinoma/2/2	8	191
			facebase RNAseq e9.5 MaxArch 2500 K4	17	1126
			Sample Type by Project: Shred 1/TCGA-Bile Duct/Cholangiocarcinoma/Intrahepatic/4/3	6	94

	Dev lower uro neuro e14.5 PelvicGanglion Sox10 top-relative-expression-ranked	14	904

Notes: All FDR & BH scores were reported as P<0.05 for results shown.

Supplemental Table 4

Differential expression analysis by PCA for co-expressed modules in rhabdomyosarcoma patients with FOXO1-PAX3/7 fusion. Statistics are reported for comparison between samples with PAX3 vs PAX7 fusion.

Module	Gene Count	PC1 Variance Explained	F	R^2	P
1	2106	0.322	1.548	0.048	0.224
2	1870	0.337	0.539	0.017	0.441
3	905	0.388	1.680	0.051	0.207
4	472	0.444	0.138	0.004	0.709
5	178	0.499	10.098	0.246	0.002
6	178	0.494	0.026	0.001	0.865
7	148	0.461	12.404	0.286	0.006
8	84	0.656	1.807	0.055	0.204
9	84	0.493	6.568	0.175	0.016
10	72	0.516	17.385	0.359	0.002
11	62	0.567	13.123	0.297	0.001
12	47	0.507	0.875	0.027	0.341
13	46	0.540	1.630	0.050	0.193
14	45	0.546	0.056	0.002	0.807
15	37	0.537	10.858	0.259	0.004
16	33	0.564	2.104	0.064	0.152
17	32	0.570	4.597	0.129	0.038
18	31	0.557	2.218	0.067	0.144
19	27	0.547	2.326	0.070	0.135
20	24	0.627	1.529	0.047	0.229
21	24	0.600	4.373	0.124	0.057
22	22	0.608	0.818	0.026	0.352
23	20	0.569	1.692	0.052	0.195
24	19	0.644	6.007	0.162	0.03
25	18	0.649	31.033	0.500	0.001
26	18	0.583	7.502	0.195	0.011
27	17	0.556	0.434	0.014	0.526
28	16	0.636	5.974	0.162	0.015
29	16	0.641	1.077	0.034	0.292
30	16	0.782	0.370	0.012	0.506
31	16	0.649	0.170	0.005	0.702
32	14	0.630	4.262	0.121	0.054
33	14	0.656	18.345	0.372	0.001
34	14	0.547	0.289	0.009	0.585
35	14	0.610	0.917	0.029	0.351
36	13	0.550	11.948	0.278	0.001
37	13	0.668	0.002	0.000	0.961
38	13	0.617	3.051	0.090	0.089
39	12	0.606	10.146	0.247	0.004
40	11	0.624	1.056	0.033	0.316
41	11	0.693	8.927	0.224	0.008
42	11	0.645	0.655	0.021	0.423
43	11	0.612	0.840	0.026	0.391
44	11	0.582	6.277	0.168	0.022

45	10	0.692	0.002	0.000	0.965
46	10	0.724	0.007	0.000	0.935
47	10	0.649	4.502	0.127	0.045
48	10	0.652	0.589	0.019	0.445
49	10	0.660	2.615	0.078	0.117
50	10	0.632	3.922	0.112	0.05
51	10	0.629	1.400	0.043	0.265
52	10	0.600	2.889	0.085	0.099
53	10	0.593	2.672	0.079	0.115

Notes: PERMANOVA was performed on distance matrix of PC1 with PAX3 or PAX7 as the explanatory variable.

Supplemental Table 5

Top 5 Co-expression Atlas (ToppFunn) results from gene list enrichment for genes that were differentially co-expressed *PAX3* versus *PAX7* fusion positive rhabdomyosarcoma.

ID	Name	Input #	Annotation #
M2012	Genes up-regulated in alveolar rhabdomyosarcoma compared to embryonic rhabdomyosarcoma	34	331
16849537-SuppTable2	Human StemCell Davicioni06	33	500
18757322-TableS3	Human Breast Creighton584	30	480
M4680	Genes up-regulated in RD cells (embryonal rhabdomyosarcoma) by expression of PAX3- or PAX7-FOXO1fusions of retroviral factors	18	225
16849537-SuppTable3	Human StemCell Davicioni389	20	319

Supplemental Figure 1

The distribution of max gene expression values per probe (A) and within gene expression variance (B) of raw data from GSE 66533. Prior to data analysis, the 33% least variant genes were removed. The subsequent distribution of max gene expression values per probe (C) and remaining gene expression variance (D) for network analysis.

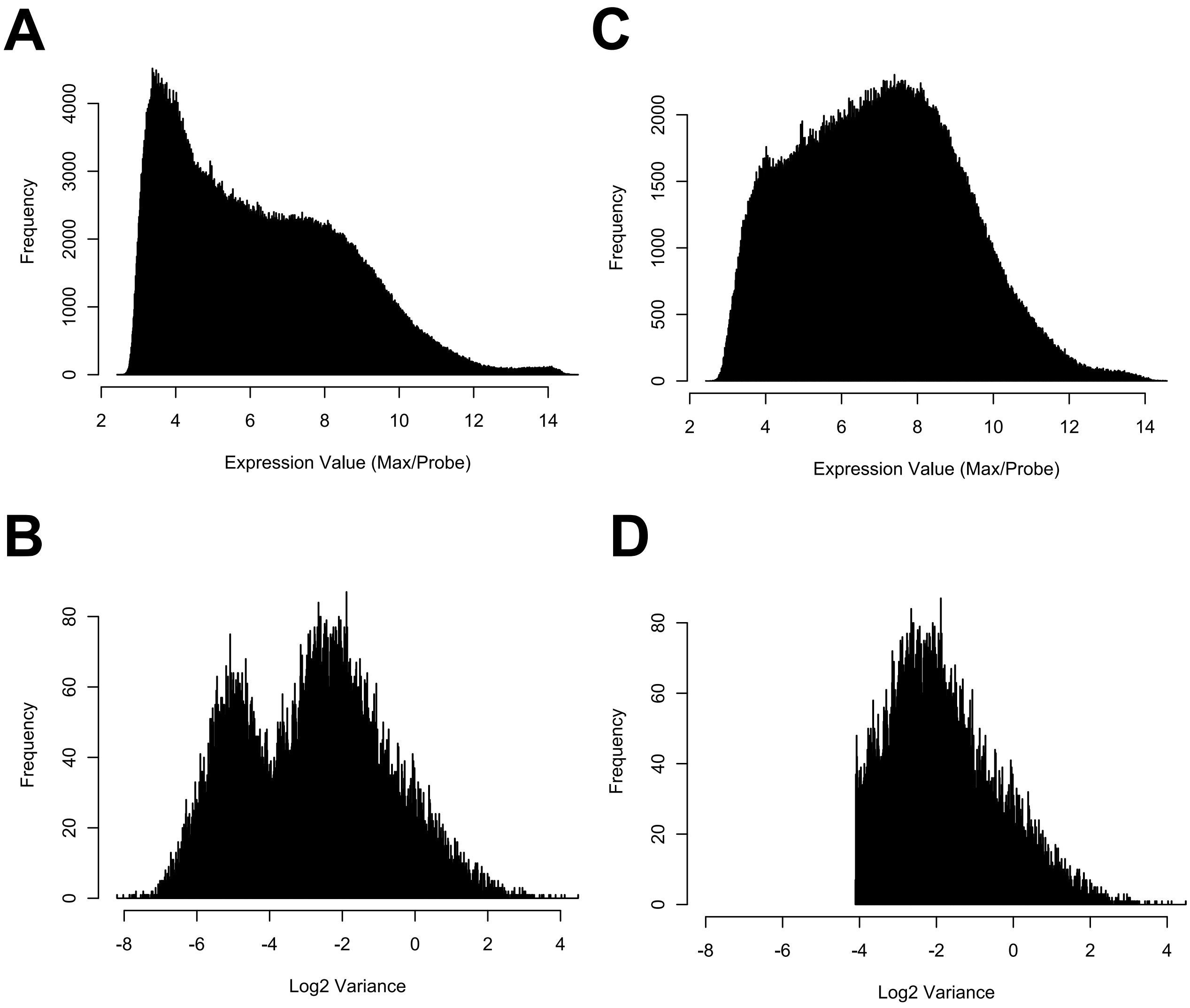

Supplemental Figure 2.

Heatmap plots of normalized gene expression values for 41 co-expression modules that were identified using lmQCM network analysis. In all plots, fusion negative samples were plotted as the leftmost 25 columns, while fusion positive samples were plotted as the rightmost 33 columns. A. Examples of co-expression modules that did not vary with respect to fusion status (A, module 1, top), that were upregulaed in fusion positive samples (A, Module 3, middle), or that were downregulated in fusion positive samples (A, module 4, bottom). B. Heatmap plots of remaining co-expression modules.

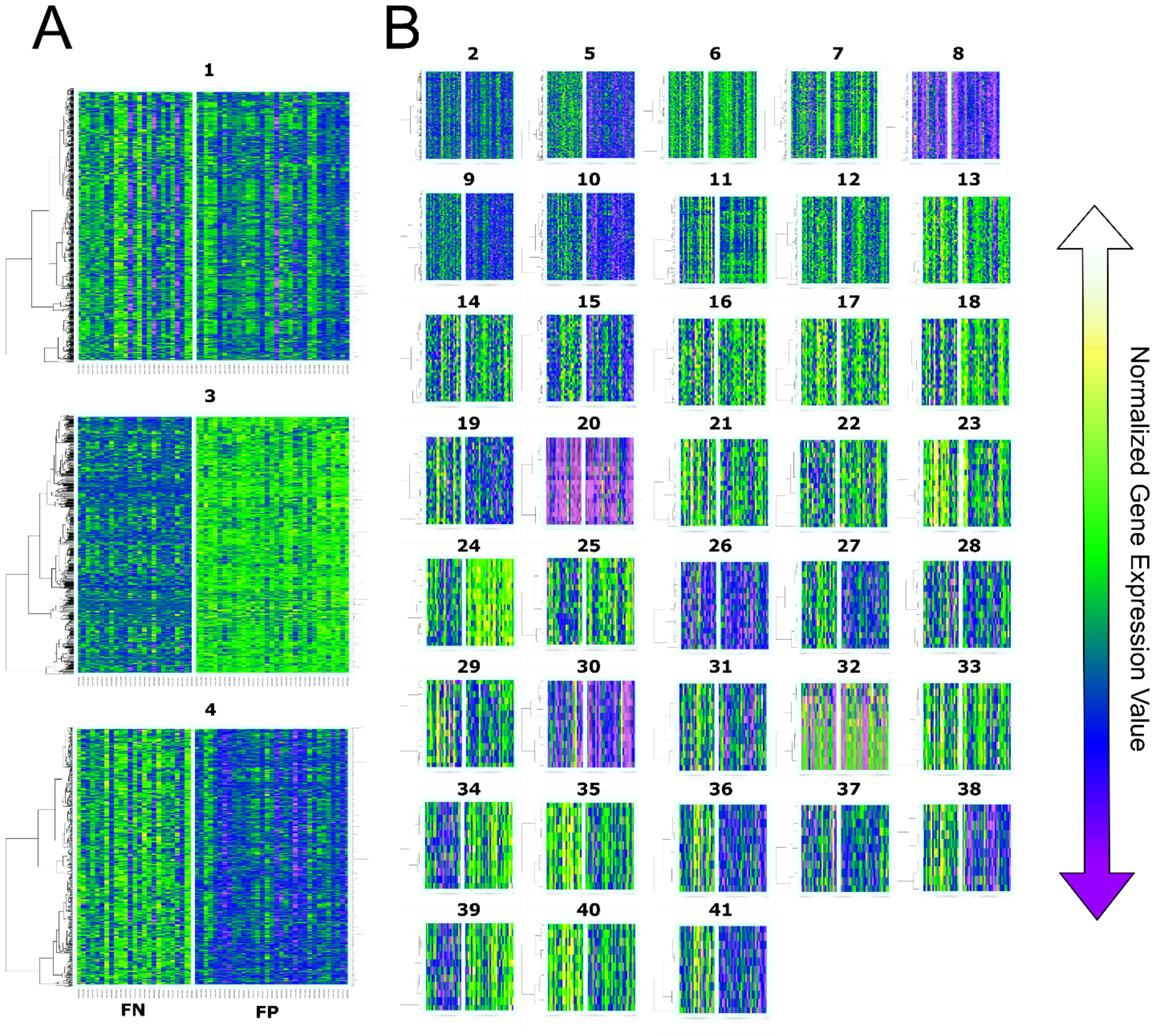

Supplement: Supplementary file 1 [file genes-10-00665-s001.pdf]
